# A Self-Powered and Highly Sensitive Flexible Contact-Pressure Sensor for Dynamic Sensing Based on Graphene-Enhanced Hydrogel

**DOI:** 10.3390/nano16080453

**Published:** 2026-04-10

**Authors:** Zhiwei Hu, Jinlong Ren, Lingyu Wan, Lin Zhang, Xuan Yang, Tao Lin

**Affiliations:** 1 Laboratory of Optoelectronic Materials and Detection Technology, School of Physical Science and Technology, Guangxi University, Nanning 530004, China; huzhiwei233@163.com (Z.H.); renjinlong@st.gxu.edu.cn (J.R.); lyw2017@gxu.edu.cn (L.W.); zl18208466213@163.com (L.Z.); 2307301165@st.gxu.edu.cn (X.Y.); 2 Guangxi Key Laboratory for Relativistic Astrophysics, School of Physical Science and Technology, Guangxi University, Nanning 530004, China; 3 Center on Nanoenergy Research, Guangxi University, Nanning 530004, China

**Keywords:** flexible contact-pressure sensor, graphene-enhanced hydrogel, self-powering, piezodielectric effect

## Abstract

A self-powered graphene-enhanced hydrogel sensor (SGHS) with high contact-pressure sensitivity and mechanical robustness was developed for precise dynamic biomechanical and material contact sensing. The device generates transient electrical signals via contact electrification and electrostatic induction during contact–separation events, eliminating the need for any external power supply. The optimized SGHS achieves a maximum peak power density of 0.23 mW·m^−2^, with contact-pressure sensitivities of 0.6 kPa^−1^ and 0.26 kPa^−1^ in the pressure ranges of 0.25–5 kPa and 5–25 kPa, respectively, which is competitive with or exceeds that of other externally powered and self-powered flexible dynamic stress sensors in the low-pressure range. Comprehensive analyses reveal that the pressure response originates from the enhanced piezodielectric effect in the graphene hydrogel layer under compression. The SGHS exhibits excellent mechanical durability, maintaining stable output after 10,000 loading–unloading cycles. Moreover, the pulse intensity, width, and waveform of its self-generated output provide distinctive features for identifying the type and surface characteristics of contacting objects. These results highlight SGHS as a promising candidate for next-generation intelligent, self-powered, and flexible dynamic sensing systems.

## 1. Introduction

In recent decades, the rapid advancement of the Internet of Things (IoT), smart wearable devices, and biomimetic robotics has dramatically increased the demand for high-performance sensors [1]. These sensors are essential for real-time health monitoring, motion tracking, and human–machine interaction. To meet the requirements of these dynamic and deformable applications, sensor components must exhibit not only high sensitivity and responsiveness but also excellent mechanical flexibility and biocompatibility. Traditional sensors based on rigid inorganic materials are inherently unsuitable for wearable applications due to their limited ability to accommodate bending, twisting, or stretching. In contrast, flexible contact-pressure sensors have emerged as a promising alternative, offering mechanical adaptability and seamless integration with soft, curved, or moving surfaces [2,3,4,5,6]. These devices have demonstrated considerable potential in applications such as electronic skin, soft robotics, and physiological signal monitoring. However, the development of flexible dynamic sensors that simultaneously achieve high sensitivity, mechanical durability, biocompatibility, environmental sustainability, and low-cost fabrication remains a significant challenge.

Typically, flexible contact-pressure sensors consist of two primary components: sensing/conductive layers and elastic substrates. Sensing/conductive layer such as MXene (Ti_3_C_2_Tx) [7,8,9,10], carbon nanotubes (CNTs) [11,12], liquid metals [13], metal nanoparticles [14,15], or conductive polymers [16,17,18,19] provide the necessary strain sensitivity to convert mechanical stimuli into electrical signals, and necessary conductivity to output the electrical signals [20,21,22,23,24,25,26]. The elastomeric substrates such as thermoplastic polyurethane (TPU) [27,28,29], polydimethylsiloxane (PDMS) [30,31,32,33], and rubber [34] provide the required stretchability and resilience. These two components are often integrated straightforward via physical adhesion like coating and printing. However, these treatments often suffer from drawbacks such as low mechanical robustness, delamination between conductive and elastic layers, and limited cyclic durability [35]. Improved techniques such as interpenetration or chemical bonding induce extra complexity and may downgrade the performance of both layers.

Among the various elastic substrates explored, hydrogels have gained substantial attention due to their soft, viscoelastic, and three-dimensional (3D) crosslinked polymer networks. Due to the porous 3D crosslinked networks of hydrogels containing abundant -OH groups, they are particularly suitable for blending with some conductive particles to form uniform “conductive hydrogels”, which combine high stretchability and conductivity, enabling strain sensing via capacitive or piezoresistive mechanisms [36]. This composite integration technique is different from the aforementioned adhesion techniques or surface bonding techniques and is superior in realizing high mechanical robustness. Furthermore, within the potential conductive fillers, MXene, graphene oxide, and carbon nanotubes offer only marginal improvements in sensor sensitivity [8,10,11,12]. Importantly, the absence of self-powering capabilities in sensors based on MXene, graphene oxide, and carbon nanotubes poses a notable challenge for their power supply systems [8,11,12]. In contrast to the above, graphene—a material known for its exceptional electrical conductivity, mechanical strength, and large surface area—has shown great potential in enhancing both the sensitivity and signal stability of hydrogel-based sensors [37,38,39]. Crucially, carbon-based materials represented by graphene possess highly tunable properties—their electron transport characteristics, surface energy, and interfacial behavior are not fixed but can be precisely tailored. Research indicates that by accurately controlling factors such as layer number, lateral size, surface functional groups, defect density (e.g., vacancies, edge structures), and 3D macro-architecture (e.g., porous foams, vertical arrays), their electrical, chemical, and mechanical properties can be finely tuned on demand, forming the core strategy of “structuralized graphene systems” [40]. This tunability constitutes the fundamental materials science rationale for our selection and optimization of graphene as the functional filler. In particular, the morphology (e.g., nanosheets, quantum dots) and defect characteristics of graphene are key to modulating its interaction with the polymer matrix, interfacial polarization, and charge transfer capability. Recent studies also highlight that the electron transport behavior of graphite/graphene structures is strongly dependent on their microstructure, providing a cutting-edge perspective for tuning the piezodielectric response of composites through material design [41].

Building upon this understanding, the specific focus of this study is to utilize pristine graphene nanosheets as fillers, aiming to construct a compressible microcapacitor network within a sodium alginate–polyacrylamide double-network hydrogel by leveraging their inherent high specific surface area, excellent electrical properties, and tunable structural characteristics. We focus on exploring the regulatory effect of graphene content on the compressive sensing performance of this composite material. However, the hydrophobicity of graphene and its tendency to agglomerate in aqueous media pose major obstacles to achieving uniform dispersion and stable device fabrication. This study aims to address this challenge through innovations in surfactant-assisted dispersion and in situ polymerization processes, thereby fabricating a high-performance, self-driven graphene-enhanced hydrogel sensor (SGHS). This sensor exhibits high contact-pressure sensitivity, rapid and accurate response, and reliable stability. Flasks are uniformly dispersed into the hydrogel matrix, which significantly improves the mechanical strength, electrical responsiveness, and output stability of the hydrogel. The resulting hydrogel is encapsulated with polydimethylsiloxane (PDMS) to form a pressure sensor that operates on a self-powered mode—the electrical signals come from the contact electrification between SGHS and the contacting object and electrostatic induction in the SGHS, with the energy collected from the mechanical motion of contact–separation, and the strength of electrical signals is modulated by the pressure-induced change in capacitance (piezodielectric effect) of the SGHS. Without any external power supply, the optimized SGHS achieves a maximum peak power density of 0.23 mW/m^2^, with pressure sensitivities of 0.6 kPa^−1^ in the range of 0.25–5 kPa and 0.26 kPa^−1^ in the range of 5–25 kPa, which is competitive with or exceeds that of other externally powered and self-powered flexible dynamic stress sensors in the low-pressure range. Moreover, due to the contact electrification in the sensing mechanism, extra information, such as the pulse width and pulse wave, can be collected for responding to object softness and roughness. It makes the SGHS highly potential in fabricating sensitive and intelligent flexible sensors in the future.

## 2. Experimental Methods

### 2.1. Design and Working Principle of SGHS

**Figure 1:**
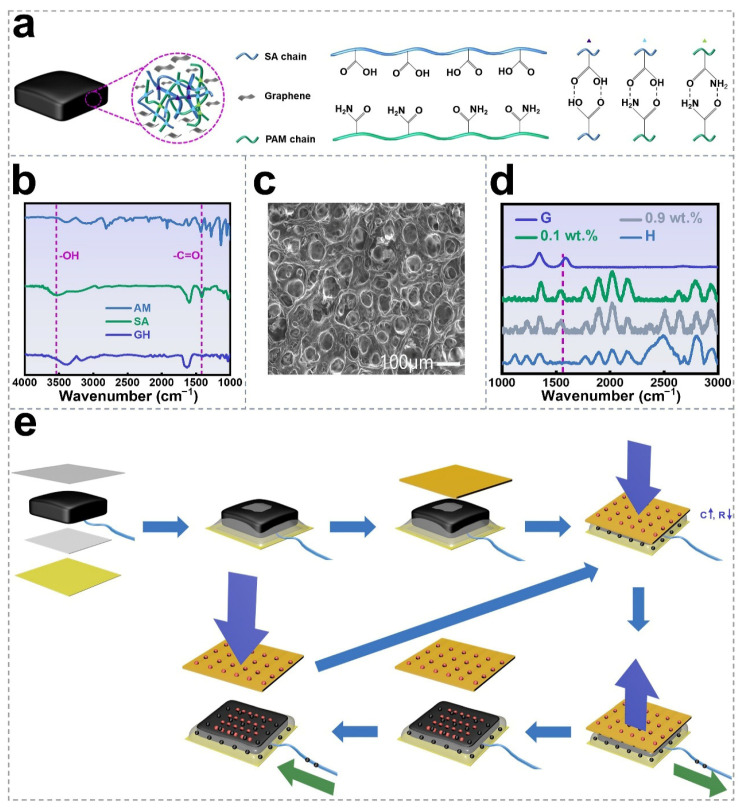
Design and working principle of SGHS: (**a**) Synthesis of GH; (**b**) FTIR spectra of AM, SA, and GH; (**c**) SEM image of GH sample (scale bar: 100 μm); (**d**) Raman spectra of graphene, GH with 0.1 wt.% graphene, GH with 0.9 wt.% graphene, and pure hydrogel; (**e**) working mechanism of SGHS.

In this study, the graphene-enhanced hydrogel (GH) was synthesized from sodium alginate (SA) and acrylamide (AM), with pristine graphene (G) serving as the composite filler to form a networked double-hydrogel structure. By carefully pretreating the graphene suspension with surfactant (the surfactant was sodium dodecyl benzene sulfonate with a content of 0.75 wt.%; see the dispersion principle and method of the G and the potential impact of the surfactant in Note S7 of Supporting Information; the effect of surfactants on conductivity and dielectric constant is an important consideration; however, due to current time constraints, we will further investigate the specific roles of different dispersants in future work). During the synthesis, AM monomers were polymerized into polyacrylamide (PAM) chains rich in -CONH_2_ groups. These PAM chains were then crosslinked with SA macromolecules, which contain -COOH groups, through intra-network interactions, leading to the formation of a dual-network hydrogel. Graphene nanosheets (graphene source: Suzhou Tanfeng Graphene Technology Co., Ltd. (Suzhou, China); graphene morphology: thickness was 3.4–8 nm, layer size was 5–50 μm, number of floors was 6 to 10 floors; as detailed in Note S6 of Supporting Information), characterized by their large specific surface area, high capacitance, stable electron mobility, and excellent chemical stability, were uniformly distributed and embedded in situ along the polymer chains, leading to the formation of a robust composite GH structure (Figure 1a). The chemical structure of the synthesized hydrogel was examined using Fourier-transform infrared (FTIR) spectroscopy. As shown in Figure 1b, the characteristic peaks of AM were observed at 3390, 1923, and 1427 cm^−1^, corresponding to the stretching vibrations of -NH_2_, -C=C, and -C=O, respectively. For SA, the peaks at 3541, 1423, and 1026 cm^−1^ were assigned to -OH, -C=O, and -C-O-C- groups, respectively. All these absorption bands were retained in the GH spectrum, confirming the successful incorporation of both SA and PAM networks. Additionally, the disappearance of individual SA and AM peaks at 3541 and 1427 cm^−1^ in the GH sample suggests the successful formation of a unified polymer network. The microstructure and graphene incorporation in GH were characterized using scanning electron microscopy (SEM) and Raman spectroscopy, respectively. As shown in Figure 1c, the freeze-dried GH (see the macroscopic and cross-sectional SEM images of the freeze-dried GH in Note S8 of Supporting Information) exhibits a well-defined, interconnected porous network, indicating that graphene incorporation enhances the compactness and flexibility of the hydrogel. The Raman spectra of GH samples with different graphene contents (0.1 wt.% and 0.9 wt.%, Figure 1d) display a characteristic peak at 1580 cm^−1^ corresponding to the G band of graphene, which is absent in the pure hydrogel. This observation confirms the successful integration of graphene into the hydrogel matrix. While SEM and Raman spectroscopy provide qualitative indicators of homogeneity, it is acknowledged that there is a lack of large-area statistical mapping data for quantitative characterization of the distribution uniformity of the nanosheets, which will be addressed in our future work.

Figure 1e illustrates the main structure and self-powered sensing principle of the SGHS assembled from GH and polydimethylsiloxane (PDMS). The GH component acts as the stress-sensitive medium, which is sandwiched with two 0.1 mm thick PDMS films. The bottom electrode is connected with the external circuit for voltage or current measurements. Upon mechanical loading, contact electrification causes charge transfer between the object and PDMS layer which is due to the difference in electron affinity: the object loses electrons and becomes positively charged, whereas the PDMS surface gains electrons and becomes negatively charged. It is worth noting that the dielectric permittivity of the GH layer is sensitive to the applied pressure as the compression densifies the polymer–water–graphene network, reduces the distance between graphene sheets, which strengthens interfacial polarization and reduces the free volume inside the hydrogel, leading to a dramatical enhancement of the permittivity of GH layer. When the pressure is released, the object is detached from the PDMS surface, leading to the separation of transferred charges and a potential decline on bottom electrode. As a result, electrostatic induction leads to an electron flow from circuit to the bottom electrode. The output voltage and current remain as pulses until the induced charges on the bottom electrode rebuild a new balance with the initially separated charges. Here the peak values of both output voltage and out current are related to the amount of initially separated charges and the capacitance of the PDMS and GH layers. Considering that the time required for the GH layer recover from the compression is long, the maximum output voltage and current are reasonable related to the variation in capacitance of GH layer, as well as the initial applied pressure. Furthermore, if the object is pressed again towards the SGHS, a reverse electron flow will be induced by the existing charges on both the PDMS and the bottom electrode. The transient response and working modes of the obtained SGHS are analyzed in detail in the subsequent section.

### 2.2. Materials and Methods

Materials: Sodium alginate (SA) powder, acrylamide (AM) powder, N,N′-methylenebisacrylamide (MBAA), and ammonium persulfate (APS) were purchased from Aladdin Reagent Co., Ltd. (Shanghai, China).

Preparation of GH: Graphene hydrogels (GHs) were prepared via in situ polymerization of SA, AM, and graphene. Graphene dispersions with mass fractions of 0.1 wt.%, 0.3 wt.%, 0.5 wt.%, 0.7 wt.%, and 0.9 wt.% (the graphene weight percentage was defined as the mass of graphene relative to the total mass of the entire precursor solution, which includes sodium alginate, acrylamide, crosslinker, initiator, deionized water, graphene and surfactant) were first prepared from graphene slurry. Subsequently, 0.4 g SA and 4 g AM were added to the dispersion, followed by ultrasonication for 10 min and magnetic stirring for 1 h until complete dissolution of the monomers. Then, 24 mg MBAA was added and stirred for 3 min, followed by the addition of 98 mg APS with another 5 min of stirring. If necessary, stirring speed and duration were increased to ensure complete mixing. The precursor solution was quickly transferred to a PTFE mold and polymerized in a water bath at 70 °C for 30 min to form GH (for instance, see the macrograph of the 0.5 wt.% GH monolith in Note S9 of Supporting Information). Despite our efforts to maintain consistent synthesis conditions, the current version of the work has not yet conducted systematic quantitative statistics on the inter-batch reproducibility of graphene dispersion homogeneity. We will prioritize this as an objective for future optimization of the preparation process.

Fabrication of flexible SGHS: GH and PDMS layers with thicknesses of 2 mm and 0.1 mm, respectively, were used to construct SGHS. Initially, a copper wire electrode was attached to GH with conductive fabric for signal output. During testing, the moving contact object was either driven by a linear motor or self-grounded to establish a reference potential. Subsequently, PDMS was evenly spread and adhered onto Kapton, GH was placed on top with the attached wire, and another PDMS layer was applied to encapsulate the structure, forming an elastic SGHS device (effective sensing area of SGHS is 2 × 2 cm^2^, see the photograph of the SGHS sample from the 0.5 wt.% GH in Note S10 of Supporting Information).

Characterization and measurements: FTIR spectra of SA, AM, and GH were recorded in the range of 600–4000 cm^−1^ at room temperature using an IRTracer-100 instrument (Shimadzu Inc., Sakyō-ku, Kyoto, Japan). The microstructure of GH was observed using a JEOL JSM-6510 scanning electron microscope (JEOL Ltd., Akishima-shi, Tokyo, Japan). Compression stress–strain tests were performed on GH samples (32 mm × 32 mm × 12 mm) using a BZ2.5/pN1S universal hardness tester at a compression rate of 5 mm/min. Contact force (see the stress value acquisition during the contact–separation testing of the SGHS in Movie S1) was digitally recorded with a DY220 pressure sensor (Bengbu Dayang Sensor System Engineering Co., Ltd., Bengbu City, Anhui, China; the minimum detectable pressure was 0.25 kPa due to the limit of the DY220 pressure sensor). For device testing, the displacement/velocity of the probe is controlled by a linear motor (LinMot E1100, NTI AG, Spreitenbach, Switzerland), thereby providing dynamic pressure input, while the contact pressure was monitored in real-time and feedback-controlled by a DY220 pressure sensor to achieve precise and repeatable loading within the range of 0.25–25 kPa; electrical signals were measured using a Keithley 6514 electrometer (Tektronix Inc., Columbus, Ohio, USA), respectively. All electrical performance tests were conducted at 24 °C and 30% RH.

## 3. Results and Discussion

### 3.1. Transient Response Analysis of SGHS

**Figure 2:**
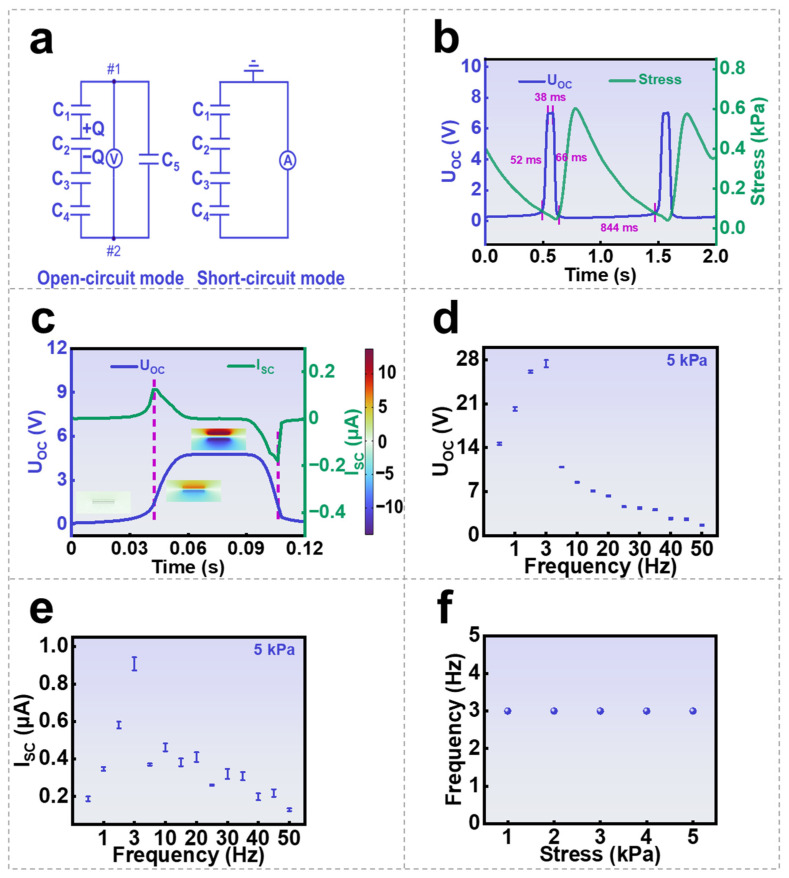
Transient Response Analysis of SGHS: (**a**) the equivalent circuits for the SGHS open-circuit mode or short-circuit mode; (**b**) the voltage response of SGHS under repetitive contact-pressure; (**c**) COMSOL simulation of the electrostatic potential in SGHS; (**d**,**e**) induced voltage and current under different frequencies at 5 kPa; (**f**) cutoff frequency of SGHS under 1, 2, 3, 4 and 5 kPa.

The working principle of a self-powered contact-pressure sensor is different from a conventional piezoresistive pressure sensor or a conventional capacitive pressure sensor as the electrostatic induction instead of external bias works as the power supply to the device. Therefore, the output voltage signal and current signal are both pulse responses to the variation in pressure during the contact instead of analog signals. This feature is essential to make an acute response to transient contacts. To examine the transient response of the SGHS, it can be set on open-circuit mode or short-circuit mode. The equivalent circuits for the two different modes are demonstrated in Figure 2a. Within the capacitors included in the circuits, C_1_ relates to the capacitance between object and the ground, C_2_ relates to the equivalent capacitance between the moving object and the top surface of PDMS, C_3_ relates to the capacitance of PDMS layer, C_4_ relates to the capacitance of GH layer, whereas C_5_ relates to the capacitance between bottom electrode and the ground. It is worth noting that, in the application scenarios of SGHS, the size and shape of the object are undefined, which means that the infinite parallel plate approximation is not applicable, and the actual electric field distribution generated by each capacitor in the system, including the fringing fields at the finite plate edges, must be taken into account. This is particularly relevant in our scenario, where the displacement of the object is often larger than the surface area of the device. Therefore, in analyzing the measurement of the open-circuit voltage ($U_{OC}$), it is necessary to introduce the concept of partial capacitance. Based on the electrostatic equilibrium of this capacitor network, the measured $U_{OC}$ should be described as (see the detailed analysis in Note S1 of Supporting Information):

(1) $$U_{OC}=\frac{C_{1}C_{3}C_{4}\;Q}{C_{1}C_{3}C_{4}C_{5}+C_{2}\;(C_{1}C_{3}C_{4}+C_{1}C_{3}C_{5}+C_{1}C_{4}C_{5}+C_{3}C_{4}C_{5})}$$

In the derivation of Equation (1), the “quasi-parallel plate” approximation was adopted to obtain an analytical expression, which has its limitations in practical point-contact or curved-surface contact scenarios. Q represents the quantity of initially separated charge between the moving object and the top surface of PDMS. It means that the object has a charge density of +Q, and the PDMS surface has a charge density of −Q, which is fixed during the contact–separation approach. Among the capacitors, the variations in $C_{1}$ and $C_{2}$ occur only in the stage after the linear motor moves the object into the “fully separated” state; therefore, $C_{1}$ and $C_{2}$ do not affect pressure sensing. $C_{3}$ and $C_{5}$ are constant. Thus, only the relationship between pressure and $C_{4}$ during the contact stage needs to be analyzed. According to the derivation in Note S1, $U_{OC}$ monotonically increases as $C_{4}$ increases. In the releasing process, $C_{1}$ and $C_{2}$ vary with the increasing separation between the object and the PDMS layer and approach a finite value when the distance becomes sufficiently large. They can be used to understand the experimental $U_{OC}$ responses measured repeatedly with the same pressure, as shown in Figure 2b, where the intrinsic electrical response time of the SGHS is 52 ms, the duration is 38 ms, the recovery time is 66 ms, and the system response time limited by mechanical loading is 844 ms—which corresponds to the mechanical movement time required for the linear motor in the test system to lift the contacting object from the “contact state” to the “fully separated state.” This time is much longer than the intrinsic electrical response time of the SGHS; therefore, it dominates the observed rising edge shape of the signal in this experimental setup, whereas the electrical response speed of the SGHS is far shorter than that. The $U_{OC}$ output signals only appear in the intervals between pressure pulses, which are associated with the releasing periods that the object does not contact with PDMS surface. The obtained $U_{OC}$ raises instantly when the applied pressure is close to zero, which indicates that the initial charge separation between the object and PDMS surface occurs, and the equivalent capacitance $C_{2}$ reduces fast due to the increase in separation distance. Considering that the charge transfer from external circuit to node #2 is not allowed in the open-circuit measurement, $U_{OC}$ finally reaches a stable maximum for the variation in system capacitance becomes very small when the separation distance is long enough. A simplified electrostatic potential simulation incorporating the boundary field effect was conducted using COMSOL Multiphysics 6.0. It is used to indicate the variation of $U_{OC}$ in one of the separation periods (Figure 2c, see the COMSOL simulation of electric potential distribution during a single contact–separation cycle in Note S11 of Supporting Information). When a pressing period starts, $U_{OC}$ decreases following with the increase in $C_{2}$ towards zero. On the other hand, SGHS exhibits a different output behavior when working on the short-circuit mode, which means that the bottom electrode can exchange charges with external circuit. As also shown in Figure 2c, only a positive pulsed current $I_{SC}$ appears at the short moment when the separation starts, which indicates an electron flow from bottom electrode to the ground, and a negative current appears when the pressing starts. The widths of the pulses of $I_{SC}$ are much shorter than the one of $U_{OC}$. It indicates that the potential of the bottom electrode cannot be maintained during the separation due to the electrostatic induction.

The frequency response of SGHS was further explored by mounting the device on a reciprocating motor. A copper foil was pressed onto a SGHS with 0.5 wt.% graphene content in GH layer as a demonstration. As shown in Figure 2d,e, both $U_{OC}$ and $I_{SC}$ raise dramatically following with increasing contact frequency in the low-frequency range (1–3 Hz). It is ascribable to the charge accumulation in fast repeated contacts [42,43], which gradually increases the initially separated charge Q, leading to the enhancements of both $U_{OC}$ and $I_{SC}$. However, the output signals suffer sudden declines when contact frequencies are beyond 3 Hz. The underlying cause for this high-frequency output decline has not been definitively determined. One possible mechanism is that air breakdown between the object and the PDMS surface leads to strong dissipation, which may restrict the maximum separable charge achievable under an ambient condition [44,45]. However, several other mechanisms also warrant serious consideration: for example, the viscoelastic hysteresis of the hydrogel layer may cause energy dissipation under high-frequency cyclic deformation; the dynamics of mechanical deformation recovery between the electrode and the active layer can affect the effective contact area and pressure; additionally, internal charge relaxation or conduction processes within the material may fail to keep pace with the frequency of external excitation at high speeds, thereby reducing the net charge output. These potential mechanisms—including the air breakdown hypothesis—require further distinction and validation through more in-depth, independent and well-controlled experiments. The systematic discrimination of these mechanisms falls beyond the scope of this preliminary investigation but represents a critical direction for future research. Interestingly, as shown in Figure 2f, within the test frequency range of 0.4–50 Hz, the peak output voltage of SGHS corresponds to a frequency of 3 Hz under different pressures of 1 kPa, 2 kPa, 3 kPa, 4 kPa, and 5 kPa. This indicates that the cutoff frequency of SGHS is independent of the applied pressure, and the applied pressure primarily affects the signal amplitude rather than the frequency response characteristics.

### 3.2. Self-Powered Sensing Performance of SGHS

**Figure 3:**
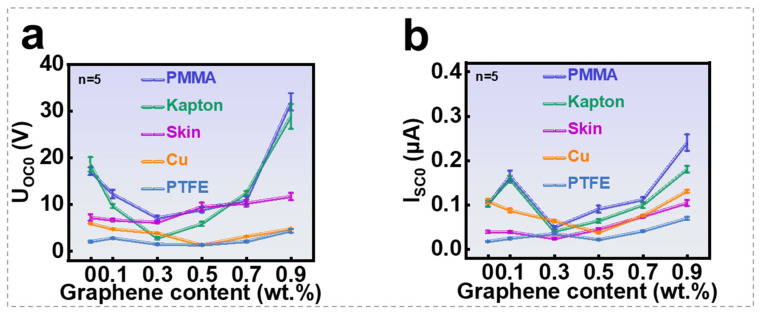
Self-powered sensing performance of SGHS: (**a**) induced voltage for different materials contacting SGHS at various graphene contents; (**b**) induced current for different materials contacting SGHS at various graphene contents.

**Figure 4:**
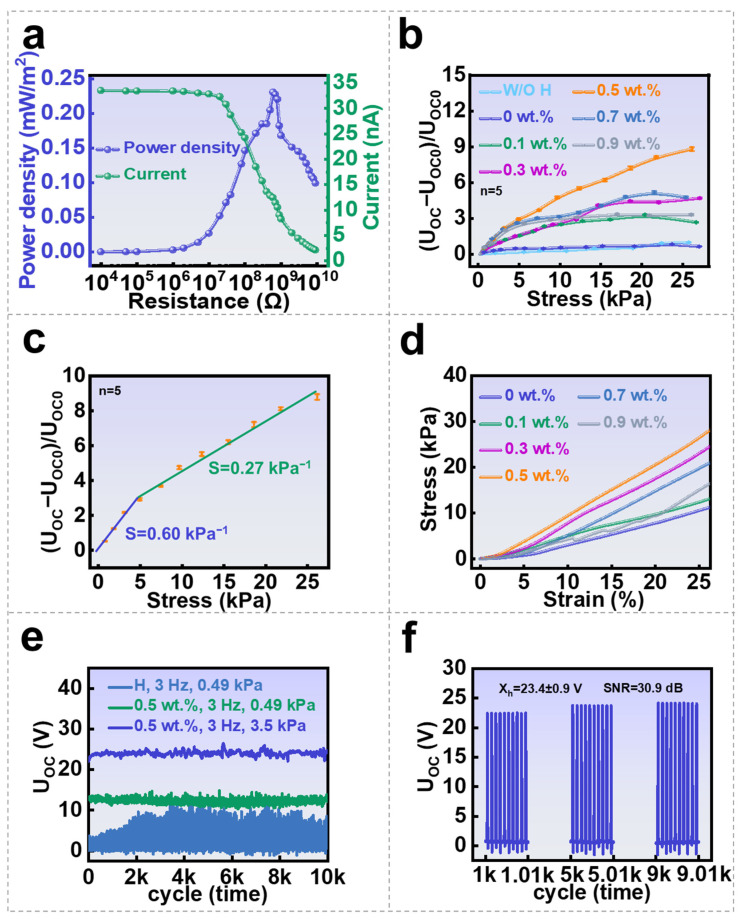
Self-powered sensing performance of SGHS: (**a**) relationship between short-circuit current, power density, and external resistance (10^4^–9 × 10^9^ Ω at 1 Hz); (**b**) voltage variation with stress for the sample without hydrogel, the pristine hydrogel sample and the SGHS samples at different graphene contents; (**c**) sensitivity of SGHS with 0.5 wt.% graphene; (**d**) the stress–strain curves obtained under compressive loads up to 28 kPa; (**e**) the pristine hydrogel device under 0.25 kPa, SGHS with 0.5 wt.% graphene under 0.25 kPa and SGHS with 0.5 wt.% graphene under 3.5 kPa were subjected to 10,000 contact–separation cycles at a frequency of 3 Hz; (**f**) statistical analysis at the 1000th, 5000th, and 9000th cycles of the cycle test (0.5 wt.%, 3 Hz, 3.5 kPa).

A group of SGHSs with different graphene contents were fabricated for sensing performance analysis. As illustrated in Figure 3a,b, SGHS generates distinct electrical responses (independent quintuplicate measurements, with detailed statistics provided in Supporting Information Note S5: Tables S1 and S2) when contacts with different materials at varying graphene contents (wt.%). At a fixed graphene concentration, the induced open-circuit voltage and short-circuit current differ across materials because of variations in electronegativity, which affect the density of triboelectric charges generated in one contact cycle. Furthermore, the output response is strongly dependent on graphene content: with increasing graphene concentration, the response first decreases and then increases. This non-monotonic trend is attributed to several competing mechanisms that are involved. At low graphene concentrations, the dispersed graphene sheets act as highly conductive but low-polarizability fillers, which restrict the dipolar orientation of polymer chains and water molecules in the hydrogel matrix. In addition, interfacial regions with reduced mobility (“bound water” or interfacial polymer layers) are formed around graphene surfaces, leading to a decrease in the overall polarization and hence a lower dielectric constant; however, as the graphene concentration increases further, the distance between graphene sheets decreases and local conductive networks begin to form. This enhances interfacial polarization (Maxwell–Wagner–Sillars polarization) and charge accumulation at graphene–matrix interfaces. Once the percolation threshold is approached, these effects dominate, resulting in a significant increase in the effective dielectric constant. To evaluate the output power, impedance matching tests were conducted using SGHS with 0.5 wt.% graphene. When connected to external resistances ranging from 10^4^ Ω to 9 × 10^9^ Ω, the maximum peak power density was observed at 6 × 10^8^ Ω, reaching 0.23 mW·m^−2^ (1 Hz contact, effective contact area 2 × 2 cm^2^, see the calculation process of peak power density in Note S2 of Supporting Information); as shown in Figure 4a, the power density and current plotted in the figure are peak values, not RMS values. This is determined by the self-powered, pulsed output nature of the SGHS, which is based on the principles of contact electrification and electrostatic induction.

To further investigate contact-pressure sensing capability of the SGHS, comparative tests were performed using hydrogel-free sample, pristine hydrogel sample, and SGHS samples with different graphene loadings under identical contact-pressure ranges. As shown in Figure 4b, the relative variation in open-circuit voltage ($U_{OC}$ − $U_{OC0}$)/$U_{OC0}$ increased with pressure enhancement (independent quintuplicate measurements, with detailed statistics provided in Supporting Information Note S5: Table S3), which verifies the contact-pressure sensing ability of the GH layers based on the fast variation in capacitance. In contrast, hydrogel-free and pristine hydrogel device exhibited much lower sensitivity compared with SGHS, which is due to the absence of polymer–water–graphene micro-capacitor structures. Within SGHS samples, sensitivity first increased and then decreased with graphene content. The optimal relative variation towards 8-fold appears at graphene concentration of 0.5 wt.%, which is related to a relatively small $U_{OC0}$ and large charge rate of capacitance value of GH layer versus contact-pressure. Excessive graphene content leads to sheet aggregation and the formation of conductive pathways throughout aggregated graphene sheets, which lead to the system capacitance no longer sensitive to the applied pressure.

The relative voltage variation versus applied pressure of SGHS with 0.5 wt.% graphene was shown in Figure 4c in detail (independent quintuplicate measurements, with detailed statistics provided in Supporting Information Note S5: Table S4). The response exhibits two distinguishable linear regions following with increase in applied pressure. The sensitivity values abstracted from the slopes are 0.60 kPa^−1^ in the low-pressure region (0.25–5 kPa) and 0.26 kPa^−1^ in the high-pressure region (5–25 kPa). In the low-pressure region, the voltage signal shows a steep increase, which is due to the dramatical increase in GH capacitance following with enhanced pressure. In the high-pressure regime, the reduction in the interlayer distance between graphene sheets approaches a limit. Therefore, the amplification of capacitance approaches saturation, leading to smaller voltage increments. To benchmark the sensing performance, the measured sensitivity of SGHS is compared with several previously reported flexible dynamic stress sensors (as detailed in Note S4 of Supporting Information). It demonstrates that the obtained SGHS exhibits comparable or superior sensitivity to these externally powered and self-powered flexible dynamic stress sensors in the low-pressure range.

The operational stability of SGHS is a critical parameter for their practical deployment in wearable and human-interactive electronics. Among the factors influencing this stability, the mechanical robustness of the hydrogel matrix plays a pivotal role. To evaluate this property, we conducted compressive mechanical testing on both pristine hydrogel sample and GH samples. The stress–strain curves obtained under compressive loads up to 28 kPa are shown in Figure 4d. Within the tested pressure range, no mechanical failure was observed for any sample, indicating that all hydrogels possess a high failure threshold. Furthermore, the slope of the stress–strain curves—indicative of compressive modulus—increased with graphene content up to 0.5 wt.% and then declined at higher concentrations. All GH samples showed superior stiffness compared to the pristine hydrogel. This trend suggests that uniformly dispersed graphene nanosheets contribute to enhanced elastic modulus and structural integrity. However, excessive graphene loading leads to sheet agglomeration and disruption of the continuous polymeric network, resulting in decreased elasticity and increased brittleness under external stress. Based on these observations, we analyzed the long-term stability of the SGHS. The pristine hydrogel device and SGHS with 0.5 wt.% graphene were subjected to 10,000 contact–separation cycles at a frequency of 3 Hz (Figure 4e). The obtained voltage signals of SGHS with or without 3.5 kPa dynamic pressure remained within a small and stable range with only minor fluctuations of ±9.5%, which may also be related to moisture variation in the GH under prolonged dynamic loading. In contrast, the signal fluctuation in the pristine hydrogel device increased rapidly during the first 2000 cycles. It verifies the view that the enhanced elastic modulus and structural integrity lead to higher sensing accuracy and stability. Furthermore, we performed a statistical analysis on the data from the 10,000 contact–separation cycle test of the SGHS with 0.5 wt.% graphene at 3 Hz and 3.5 kPa (the detailed statistics provided in Supporting Information Note S5: Table S9). Analysis at key cycle nodes—specifically the 1000th, 5000th, and 9000th cycles—yielded the following results: the peak voltage was 23.4 ± 0.9 V, and the signal-to-noise ratio (SNR) was 30.9 dB, as shown in Figure 4f. This further demonstrates the stable and reliable output characteristics of the SGHS, though it remains subject to its inherent hysteresis behavior.

### 3.3. Sensing Mechanism of SGHS

In the preceding sections, we have ruled out the influence of frequency response characteristics on the operational mechanism of the SGHS. Nevertheless, prior studies suggest that other mechanisms may also contribute to the dynamic pressure sensitivity of contact-electrification-based systems. For example, higher applied pressure can improve interfacial contact, resulting in greater initial charge separation density. In addition, variations in applied pressure can alter the resistance of the GH layer; reduced resistance may facilitate larger leakage currents between the PDMS layer and the bottom electrode, thereby decreasing the initial charge density. Variations in applied pressure may also modify the geometric capacitance and pressure-dependent dielectric capacitance of the GH layer. To clarify the dominant sensing mechanism of the SGHS and exclude these possible influences, we performed both piezoresistive and piezodielectric characterizations on the standalone GH structures.

**Figure 5:**
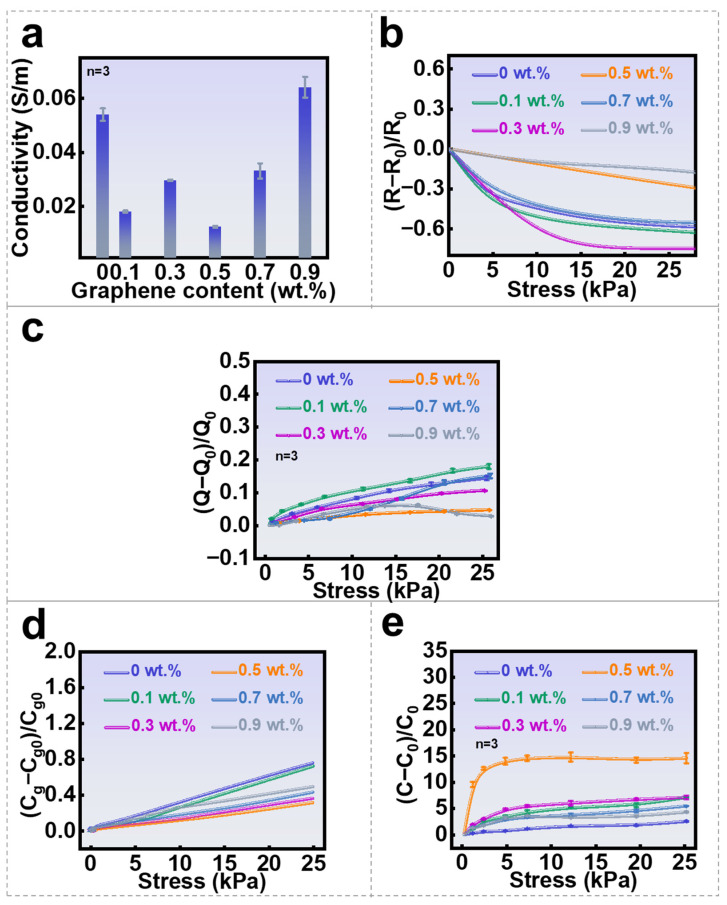
Sensing mechanism of SGHS: (**a**) the conductivity of hydrogels; (**b**) the relationship between the variations in resistance and contact-pressure for different hydrogels at 10 V; (**c**) the relationship between the induced electrical variations and pressure for different SGHS samples under the incipient contact–separation mode; (**d**) the relationship between capacitance variations caused by hydrogel thickness and contact-pressure for different SGHS samples; (**e**) the relationship between the variations in total capacitance and contact-pressure for different SGHS samples.

As shown in Figure 5a, the intrinsic conductivities of the GH samples without applied pressure fall within the weakly conductive range of 0.02–0.06 S·m^−1^ (use a two-electrode method with copper sheets as the electrode material, measurements were conducted under pressure-free static conditions, the geometric dimensions of the test sample were 1.5 cm × 1.0 cm × 2.5 cm; independent triplicate measurements, with detailed statistics provided in Supporting Information Note S5: Table S5). Interestingly, low-density graphene doping slightly decreases the conductivity of the pristine hydrogel. This phenomenon arises because the dispersed graphene nanosheets fail to form continuous conductive networks; rather, the isolated flakes disrupt the ionic conduction channels that primarily govern the conductivity of the pristine hydrogel. Moreover, graphene incorporation reduces the free water content, increases interfacial resistance, and subtly modifies the porous microstructure, all of which impede ion mobility. With further increases in graphene concentration, however, the overall conductivity gradually rises, implying the formation of interconnected conductive pathways among the graphene sheets. Figure 5b presents the resistance variation in GH layers as a function of applied pressure. The observed piezoresistive response does not correlate with the relative variation in open-circuit voltage shown in Figure 4b. Specifically, the relative change in resistance (0–0.75) is approximately an order of magnitude smaller than the corresponding voltage variation. Furthermore, while the pristine hydrogel exhibits significantly lower pressure sensitivity in open-circuit voltage compared to the 0.5 wt.% GH sample, it shows higher piezoresistive sensitivity. These observations collectively demonstrate that the piezoresistive effect is not the dominant sensing mechanism governing SGHS behavior. Theoretically, increased conductivity of the GH layer may contrarily lead to higher leakage current throughout the GH layer, which is disadvantageous in maintaining the initially separated charges. However, considering that the intrinsic conductivities of GH layers are pretty low, and the insulative PDMS layer further suppresses the leakage current, this adverse effect is not obvious in our case.

Figure 5c illustrates the relative variation in initially separated charge as a function of applied pressure (independent triplicate measurements, with detailed statistics provided in Supporting Information Note S5: Table S6). The results reveal that the separated charge quantity increases with higher pressure, attributable to improved contact between the object and the PDMS surface. This effect becomes less pronounced as graphene concentration rises from 0.1 wt.% to 0.5 wt.%, owing to the increased elastic modulus of the GH network. Consequently, softer samples promote more efficient charge separation. However, because the open-circuit voltage should scale proportionally with the amount of separated charge, the experimentally observed voltage behavior contradicts this expectation. Hence, enhanced surface contact is not the primary contributor to the SGHS’s sensing response.

Since the total capacitance change in GH is a combined result of geometric effects (thickness) and intrinsic material property changes (dielectric constant), it is necessary to first examine the capacitance change caused solely by variations in GH thickness. Assuming the dielectric constant ε and material volume V remain constant, the thickness reduction ratio $\frac{\Delta d}{d_{0}}$ due to compression approximates the strain value σ. Consequently, the capacitance change induced purely by thickness reduction can be estimated using the formula $C_{g}=\varepsilon \frac{V}{{d_{0}}^{2}(1-\sigma )}$. By utilizing the stress–strain data from Figure 4e, the stress-dependent capacitance change ($C_{g}$) resulting only from GH thickness variation is calculated and plotted in Figure 5d. The results show that the increase in $C_{g}$ with rising contact pressure is minimal. The maximum capacitance enhancement is merely 0.76-fold, observed in the undoped hydrogel sample, which stems from the significant influence of its inherent softness on capacitance through thickness variation. Next, the total capacitance change in the GH is measured, and its relation to the capacitance change from thickness variation is analyzed to determine the contribution ratio of the piezodielectric effect to the total GH capacitance. Figure 5e presents the direct measurement results of the total capacitance (C_0_) for the applied GH layer (under a DC voltage of 30 V, the GH sample was clamped between two parallel plate electrodes, the stored charge at different static pressures was measured using a Keithley 6514 electrometer, allowing for the calculation of C_0_; independent triplicate measurements, with detailed statistics provided in Supporting Information Note S5: Table S7). The results clearly demonstrate a significant monotonic increase in C_0_ with rising contact pressure. This characteristic is more pronounced in GH samples than in pure hydrogel. Particularly, the 0.5 wt.% GH sample exhibits the optimal capacitance enhancement, up to 15-fold. Analysis combining Figure 5d,e reveals that in the low-pressure region (0.25–5 kPa), the contribution of the geometric capacitance $C_{g}$ to the total capacitance C_0_ increase is less than 24% for undoped hydrogel (see the statistical table of the relationship between the contribution proportion of capacitance change due solely to thickness and the contribution proportion of capacitance change due solely to the piezoelectric effect in Note S3 of Supporting Information), with the piezodielectric effect contributing over 75%. For graphene-doped hydrogel, $C_{g}$ contributes less than 5%, while the piezodielectric effect contributes over 95%. This strongly demonstrates that within this high-sensitivity range, the sharp capacitance rise primarily originates from enhanced dielectric polarization due to compression of the graphene hydrogel composite network, rather than mere geometric thinning. In the high-pressure region (5–25 kPa), the proportion of the geometric effect increases, while the enhancement of the dielectric constant tends to saturate. This characteristic aligns well with and validates the high pressure sensitivity of the output voltage observed in the low-pressure (0.25–5 kPa) range in Figure 4c.

### 3.4. Response of the SGHS to Object Softness and Roughness

**Figure 6:**
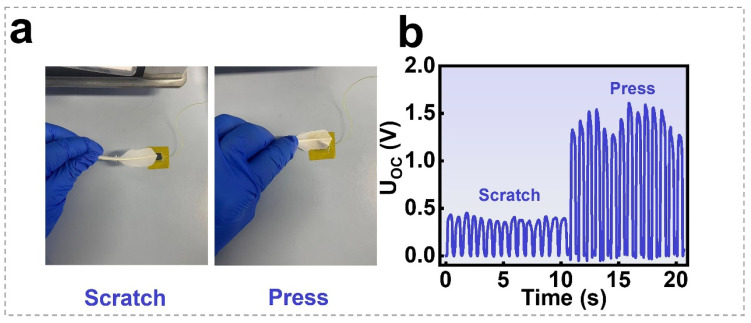
Identification to the type of contact object using SGHS: (**a**,**b**) the characteristic open-circuit voltage ($U_{OC}$) responses of the 0.5 wt.% SGHS to gentle mechanical scratch and compression using a feather.

Figure 6a,b illustrates the characteristic open-circuit voltage ($U_{OC}$) responses of the 0.5 wt.% SGHS composite under gentle mechanical contact and compression applied via a feather (see the $U_{OC}$ signal generated during feather-based scratch and dynamic pressure application on the SGHS in Movie S2 and Movie S3). It can be observed that the SGHS effectively detects feather scratch, and exhibits distinct output responses depending on the contact force—whether from light touch or pressing—demonstrating its high sensitivity and discriminative capability in contact-pressure detection.

**Figure 7:**
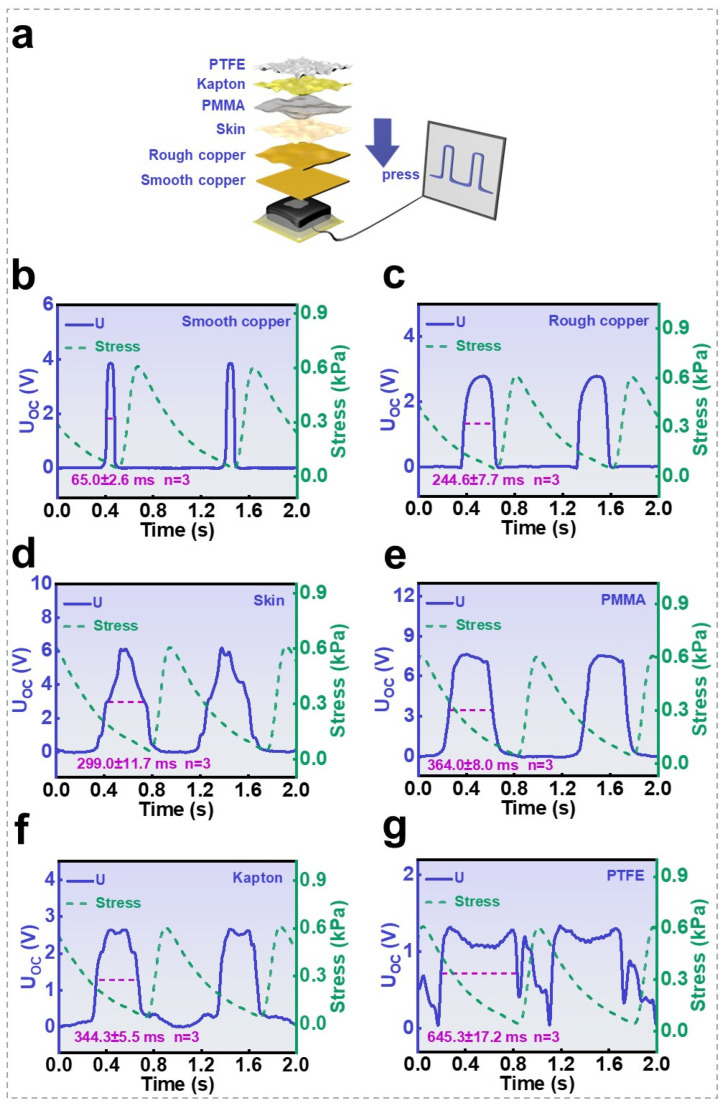
Preliminary extraction and analysis of tactile features using SGHS: (**a**) the schematic diagram of the contact electrification of different objects with the sensor surface; (**b**–**g**) the characteristic open-circuit voltage ($U_{OC}$) responses of the 0.5 wt.% SGHS when contacting various objects under a pulsed pressure of 0.6 kPa.

According to the operating principle of the SGHS, the pulse-like output signals originate from contact electrification between the object and the sensor surface (Figure 7a). Our findings demonstrate that the signal strength depends on both the electronegativity of the contacting material and the applied pressure. Consequently, the type of contact object cannot be distinguished solely based on signal intensity. However, the acquired electrical signals also contain additional information correlated with the object’s physical characteristics, offering the potential to identify the object type in conjunction with signal amplitude analysis.

Figure 7b–g display the characteristic open-circuit voltage ($U_{OC}$) responses of the 0.5 wt.% SGHS when contacting various objects under a pulsed pressure of 0.6 kPa. As indicated in each figure, identical pressure waveforms were applied across all experiments, resulting in stable and repeatable pulse-shaped $U_{OC}$ outputs. Notably, beyond variations in pulse amplitude, the waveform morphology also exhibits a clear correlation with the type of contact object. A clear trend can be extracted from these observations: the pulse width increases when the SGHS interacts with softer or rougher surfaces. Specifically, when a hard and smooth object—such as a polished copper plate—contacts the SGHS, sharp $U_{OC}$ pulses occur precisely at the moment when the applied pressure returns to zero. Conversely, when either a rougher surface of the same material or a softer object makes contact, the $U_{OC}$ pulse extends into the pressure-release stage, resulting in a broader pulse width. This phenomenon becomes increasingly pronounced as the contacting surface becomes softer or rougher. The underlying reason may lie in the partial separation between the SGHS surface and the object. For hard and smooth surfaces, complete charge separation occurs rapidly, yielding short, sharp pulses. In contrast, for softer or rougher surfaces, localized detachment begins before the global pressure fully relaxes to zero, producing earlier signal initiation and an extended pulse duration. Therefore, the output pulse width serves as a reliable indicator of the contacting surface’s hardness and roughness (independent triplicate measurements, with detailed statistics provided in Supporting Information Note S5: Table S8). Moreover, the surface characteristics influence not only pulse width but also waveform morphology. For example, contact with human skin produces a convex waveform (Figure 7d), whereas contact with polytetrafluoroethylene (PTFE) yields a concave waveform (Figure 7g). These waveform variations likely arise from differences in surface adhesion and elastic recovery among the contacting materials. Highly adhesive materials, such as skin, exhibit slower detachment during pressure release, resulting in gradually decaying, convex-shaped pulses. Conversely, more elastic materials, such as PTFE, tend to rebound upon unloading, producing transient reverse discharges that manifest as concave waveforms. Although the microscopic mechanisms underlying these behaviors are complex, this complexity does not preclude data-driven analysis. The pulse signals acquired from flexible pressure sensors exhibit width and shape characteristics closely associated with the material, roughness, and hardness of the contacting object, thereby enabling the system to respond to object softness and roughness. This provides the potential for multimodal object recognition and lays a potential feature foundation for future machine learning-based object identification—this will also be the core focus of our subsequent research.

## 4. Conclusions

In summary, a highly sensitive, self-powered SGHS for dynamic pressure sensing was successfully fabricated through the incorporation of pristine graphene nanosheets into a sodium alginate-polyacrylamide dual-network hydrogel. The synergistic combination of the hydrogel’s viscoelastic network and graphene’s conductive properties enables the SGHS to efficiently convert mechanical stimuli into electrical signals through contact electrification and electrostatic induction. The optimized device delivers a maximum peak power density of 0.23 mW·m^−2^ and remarkable pressure sensitivities of 0.6 kPa^−1^ (0.25–5 kPa) and 0.26 kPa^−1^ (5–25 kPa). Its performance in the low-pressure range is competitive with or surpasses that of other externally powered and self-powered flexible dynamic stress sensors. The sensor demonstrates long-term stability, mechanical resilience, and reproducible output under repeated cycling. Detailed investigations confirm that its sensing behavior mainly arises from the pressure-dependent dielectric modulation of the graphene hydrogel layer. Furthermore, the unique correlation between pulse waveform characteristics and surface properties of contacting materials provides a new approach for contact object recognition, suggesting the feasibility of machine-learning-based classification. Overall, the SGHS represents a sustainable and multifunctional platform capable of responding to the softness and roughness of contact objects, with the potential for multimodal object recognition. It thereby provides a potential foundation for subsequent applications in areas such as dynamic signal monitoring in wearable electronics, tactile feedback for soft robotics, and human–machine interaction.

## Data Availability

All data used in this study are available upon request from the corresponding author.

## Supplementary Materials

The following supporting information can be downloaded at: https://www.mdpi.com/article/10.3390/nano16080453/s1.
