# Supplementary material for: A Self-Powered and Highly Sensitive Flexible Contact-Pressure Sensor for Dynamic Sensing Based on Graphene-Enhanced Hydrogel
Source: Nanomaterials (Basel). 2026 Apr 10;16(8):453. doi: 10.3390/nano16080453 (PMC13119002; doi:10.3390/nano16080453)

Supporting information
Supporting formula

Note S1: The U_{OC} mentioned in the article refers to the V_e, and the capacitance of GH refers to the C_4, The analysis of the equation relationship between the V_e and the C_4.

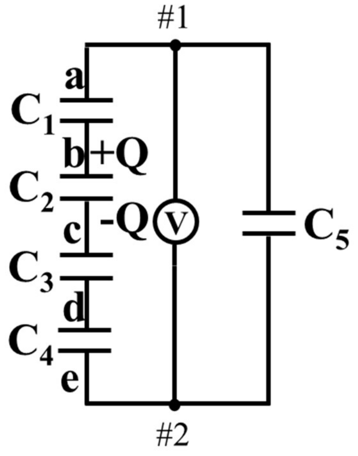

The equivalent circuit diagram：

The C_1 is the capacitance between the object and the ground;

The C_2 is the capacitance between the object and PDMS;

The C_3 is the capacitance of PDMS;

The C_4 is the capacitance of GH;

The C_5 is the capacitance between the bottom of GH and the ground.

View the equivalent circuit as several nodes. Label the ends of C_1, C_2, C_3, and C_4 from top to bottom as **a, b, c, d,** and **e**. Take node **a** at the top as the zero potential. The nodes are connected by capacitors: C_1 connects **a-b**, C_2 connects **b-c**, C_3 connects **c-d**, C_4 connects **d-e**, and C_5 is between **a** and **e**. Starting from the node electrostatic equilibrium equation, finally calculate the bottom potential V_e (that is, the reading of the voltmeter, relative to the top node **a**), and explore the relationship between V_e and C_4.

Initially, only the two plates of C_2 carry equal but opposite charges of $\pm Q$, which is equivalent to the node charges

$q_a = q_d = q_e = 0,\quad q_c = -Q,\quad q_b = +Q$;(The total charge of each conductor remains unchanged in the absence of conducting paths).

From the node equation of capacitance $\sum_j C_{ij}\,(V_i - V_j) = q_i$ (electrostatic equilibrium of the capacitance network, holds for each node),solve for the bottom potential $\boldsymbol{V_e}$ (the reading of the voltmeter is $\boldsymbol{V_e}$-$\boldsymbol{V_a}$ = $\boldsymbol{V_e}$). Algebraic solution gives:

$$V_e = -\frac{C_1C_3C_4}{D}\,Q$$

The denominator $D = C_1C_2C_3C_4 + C_1C_2C_3C_5 + C_1C_2C_4C_5 + C_1C_3C_4C_5 + C_2C_3C_4C_5$.

Express $\boldsymbol{U_{OC}}$ in terms of the absolute value of $\boldsymbol{V_e}$:

$$U_{OC} = |V_e| = \frac{C_1C_3C_4\,Q}{C_1C_3C_4C_5 + C_2\,(C_1C_3C_4 + C_1C_3C_5 + C_1C_4C_5 + C_3C_4C_5)}.$$

Taking the derivative of $\boldsymbol{C_4}$, yields:

$$\frac{\partial U_{OC}}{\partial C_4} = \frac{C_1C_3Q\cdot(C_1C_2C_3C_5)}{(\mathrm{D})^2} > 0,$$

(both the numerator and denominator are positive), thus $\boldsymbol{U_{OC}}$ monotonically increases as $\boldsymbol{C_4}$ increases.

Conclusion: In the device, only $\boldsymbol{C_2}$ is charged, and it creates an electric potential difference along the series capacitor chain. Changing $\boldsymbol{C_5}$ will alter the capacitance coupling between the nodes, causing the voltages at each node to be redistributed (but the net charge on each isolated conductor remains unchanged), thereby causing a change in the voltages at both ends (top and bottom). The mathematical results show that in the presence of parallel capacitor $\boldsymbol{C_5}$, increasing $\boldsymbol{C_4}$ will increase $\boldsymbol{U_{OC}}$; if $\boldsymbol{C_5}$ is exactly zero, then $\boldsymbol{U_{OC}}$ will always be equal to $\boldsymbol{Q/C_2}$, independent of $\boldsymbol{C_4}$.

Note S2: The calculation process of peak power density.

Peak power density $P_d = \frac{I^2 R}{A}$, I is the peak output current, R is the load resistance, A is the effective contact area (4×10^{-4} m^2). When the load resistance is 6×10^8 Ω, the peak current is 12.4 nA, the peak voltage U is 7.44 V, and the instantaneous power density P_d is 0.23 mW/m^2.

Note S3: Statistical table of the relationship between the contribution proportion of capacitance change due solely to thickness (T_{Cg}) and the contribution proportion of capacitance change due solely to the piezoelectric effect (T_{Cd}) within the total capacitance change (C_0) in GH.

	**0.49-5 kPa**		**5-25 kPa**	
	**T_{Cg}**	**T_{Cd}**	**T_{Cg}**	**T_{Cd}**
**0 wt.%**	24.6%	75.4%	34.1%	65.9%
**0.1 wt.%**	3.8%	96.2%	10.0%	90.0%
**0.3 wt.%**	1.5%	98.5%	5.7%	94.3%
**0.5 wt.%**	0.4%	99.6%	2.0%	98.0%
**0.7 wt.%**	3.6%	96.4%	7.9%	92.1%
**0.9 wt.%**	4.6%	95.4%	13.3%	86.7%

Note S4: Comparative statistical table of similar dynamic stress sensors' performance and discussion on normalization.

**Document number**	**Sensing mechanism**	**Pressure range and sensitivity**	**Device structure**	**Test conditions and response time**
**[10]**	triboelectric; piezoresistive; resistive output	(0–120 kPa); 0.0164 kPa^{-1}(0–16 kPa), 0.00286 kPa^{-1}(16–120 kPa)	Mxene/PVA/CP/GO hybrid structure	self-powered, tensile stress；168 ms
**[11]**	piezoresistive; resistive output	(0.05–6.3 kPa); 0.31 kPa^{-1}(0.05–3.8 kPa), 0.03 kPa^{-1}(3.8-6.3 kPa)	CNT/GO@PDMS dual-sublayer structure	external power supply, tangential compressive stress；—
**[12]**	triboelectricity; piezoresistive; resistive output	(0–10 kPa); 0.127 kPa^{-1}(0–5 kPa), 0.04 kPa^{-1}(5-10 kPa)	PVA/CNT/G island-bridge structure	self-powered, tensile stress；—
**[17]**	triboelectricity; capacitive; capacitive output	(0–4 kPa); 0.75 kPa^{-1}(0–4 kPa)	PVA/CNF-PE-PVA/CNF three-layer structure	self-powered, tensile stress；—
**[19]**	triboelectricity; piezoresistive; voltage output	(1–10 kPa); 0.24 kPa^{-1}(1–10 kPa)	PPy@CT single-electrode structure	self-powered, compressive stress；—
**[33]**	triboelectricity; piezoresistive; voltage output	(1.25–62.5 kPa); 0.074 kPa^{-1}(1.25–62.5 kPa)	FEP/PDMS porous structure	self-powered, compressive stress；—
**[36]**	piezoresistive; resistive output	(0–28 kPa); 0.038 kPa^{-1}(0–16.5 kPa), 0.008 kPa^{-1}(16.5-28 kPa)	silicon nanofiber reinforced hydrogel structure	external power supply, compressive stress；—
**This Work**	triboelectricity; capacitive; voltage output	(0–25 kPa); 0.6 kPa^{-1}(0–5 kPa), 0.26 kPa^{-1}(5-25 kPa)	PDMS-GH-PDMS three-layer structure	self-powered, compressive stress；52 ms

Continue Note S4: Normalization discussion.

Since the sensor output signals are resistance signals in references **[10]**, **[11]**, **[12]**, **[36]**, the sensitivity $S = \delta(\Delta R/R_0)/\ \delta P$.

Since the sensor output signals are capacitance signals in references **[17]**, the sensitivity $S = \delta(\Delta C/C_0)/\delta P$

Since the sensor output signals are voltage signals in references **[19]**, **[33]** and this work, the sensitivity $S = \delta(\Delta U/U_0)/\delta P$.

a. Classifying based on the classification by triboelectric mechanism, the sensors in references **[10]**, **[12]**, **[17]**, **[19]**, and **[33]** are categorized as externally powered sensors. In contrast, the sensor developed in this work is a self-powered sensor, offering a distinct advantage in terms of power supply.

b. Classifying based on the type of test force in the test conditions, the sensors in references **[11]**, **[19]**, **[33]**, **[36]**, and this work are categorized as compressive stress sensors. Thus, the sensitivity $S = \delta(\Delta U/U_0)/\delta P$ or $\delta(\Delta R/R_0)/\delta P$, and the results clearly show that the sensitivity of the sensor in this work ranks **first** in the low-pressure range (1.25–6.3 kPa).

c. Classifying based on the sensing mechanism (two out of three types matching), pressure range, and sensitivity, the sensors in references **[17]**, **[19]**, **[33]**and this work fall into the same category. Thus, the sensitivity $S = \delta(\Delta U/U_0)/\delta P$ or $\delta(\Delta C/C_0)/\delta P$, and the results clearly show that the sensitivity of the sensor in this work ranks **first** in the low-pressure range (1.25–10 kPa).

In summary, compared to other flexible sensors that require an external power source and self-powered flexible sensors, the sensor developed in this work demonstrates a leading sensitivity, a moderate pressure range, and a relatively straightforward device structure.

Note S5: Statistical analysis of reproducibility for key data, including device performance, material properties, preliminary contact identification experiments, and 10,000-cycle testing.

Table S1: Analysis of output voltage diagram reproducibility with varying materials/graphene content (n=5).

	PMMA	Kapton	Skin	Cu	PTFE
0 wt.%	17.14±0.84 V, RSD=4.9%	18.58±1.56 V, RSD=8.4%	7.2±0.69 V, RSD=9.6%	5.91±0.04 V, RSD=0.7%	2.04±0.18 V, RSD=8.8%
0.1 wt.%	12.22±0.93 V, RSD=7.6%	9.66±0.43 V, RSD=4.5%	6.68±0.28 V, RSD=4.2%	4.73±0.03 V, RSD=0.6%	2.80±0.11 V, RSD=3.9%
0.3 wt.%	7.12±0.47 V, RSD=6.6%	2.78±0.27 V, RSD=9.7%	6.18±0.19 V, RSD=3.1%	3.87±0.03 V, RSD=0.8%	1.49±0.12 V, RSD=8.0%
0.5 wt.%	8.86±0.56 V, RSD=6.3%	5.84±0.45 V, RSD=7.7%	9.52±0.88 V, RSD=9.2%	1.31±0.01 V, RSD=0.8%	1.33±0.12 V, RSD=9.0%
0.7 wt.%	10.90±0.83 V, RSD=7.6%	12.48±0.39 V, RSD=3.1%	10.26±0.70 V, RSD=6.8%	3.14±0.02 V, RSD=0.6%	2.08±0.19 V, RSD=9.1%
0.9 wt.%	31.98±1.85 V, RSD=5.8%	28.86±2.64 V, RSD=9.1%	11.70±0.76 V, RSD=6.5%	4.77±0.04 V, RSD=0.8%	4.36±0.40 V, RSD=9.2%

The relative standard deviation (RSD) for all the data points mentioned above is less than 10.0%, demonstrating good repeatability and stability.

Continue Note S5: Table S2: Analysis of the reproducibility of output current plots under different material/graphene contents (n=5).

	PMMA	Kapton	Skin	Cu	PTFE
0 wt.%	0.099±0.004 uA, RSD=4.0%	0.103±0.006 uA, RSD=5.8%	0.038±0.003 uA, RSD=7.9%	0.108±0.005 uA, RSD=4.6%	0.017±0.001 uA, RSD=5.9%
0.1 wt.%	0.165±0.012 uA, RSD=7.3%	0.158±0.007 uA, RSD=4.4%	0.039±0.002 uA, RSD=5.1%	0.087±0.004 uA, RSD=4.6%	0.024±0.002 uA, RSD=8.3%
0.3 wt.%	0.049±0.003 uA, RSD=6.1%	0.040±0.002 uA, RSD=5.0%	0.023±0.002 uA, RSD=8.7%	0.063±0.002 uA, RSD=3.2%	0.035±0.002 uA, RSD=5.7%
0.5 wt.%	0.090±0.008 uA, RSD=8.9%	0.063±0.004 uA, RSD=6.3%	0.044±0.003 uA, RSD=6.8%	0.037±0.002 uA, RSD=5.4%	0.021±0.001 uA, RSD=4.8%
0.7 wt.%	0.111±0.006 uA, RSD=5.4%	0.099±0.006 uA, RSD=6.1%	0.073±0.003 uA, RSD=4.1%	0.075±0.001 uA, RSD=1.3%	0.040±0.002 uA, RSD=5.0%
0.9 wt.%	0.240±0.018 uA, RSD=7.5%	0.18±0.007 uA, RSD=3.9%	0.104±0.006 uA, RSD=5.8%	0.131±0.003 uA, RSD=2.3%	0.069±0.003 uA, RSD=4.3%

The relative standard deviation (RSD) for all the data points mentioned above is less than 10.0%, demonstrating good repeatability and stability.

Continue Note S5: Table S3: Repeatability analysis of the graphical representation of output voltage relative changes under different graphene content/pressure conditions (n=5).

W/OH	2.08 kPa: 0.085±0.008 , RSD=9.4%；5.61 kPa: 0.166±0.009 , RSD=5.4%; 7.60 kPa: 0.299±0.015 , RSD=5.0%; 10.80 kPa: 0.267 ± 0.015 , RSD=5.6%; 14.21 kPa: 0.484 ± 0.013 , RSD=2.7%; 18.94 kPa: 0.493 ± 0.015 , RSD=3.0%;22.44 kPa: 0.921±0.033 , RSD=3.6%; 25.77 kPa: 0.980±0.008 , RSD=8.2%
0 wt.%	0.78 kPa: 0.166±0.007 , RSD=4.2%；1.88 kPa: 0.296±0.01 , RSD=3.4%; 2.77 kPa: 0.381±0.014 , RSD=3.7%; 4.58 kPa: 0.477±0.009 , RSD=1.9%; 5.71 kPa: 0.481±0.011 , RSD=2.3%; 8.01 kPa: 0.452±0.009 , RSD=2.0%; 10.09 kPa: 0.556±0.010 , RSD=1.8%; 13.21 kPa: 0.580±0.008 , RSD=1.4%; 15.24 kPa: 0.680±0.015 , RSD=2.2%; 19.13 kPa: 0.649±0.012, RSD=1.8%; 23.74 kPa: 0.834±0.013 , RSD=1.6%; 27.07 kPa: 0.635±0.012 , RSD=1.9%
0.1 wt.%	0.81 kPa: 0.471±0.009 , RSD=1.9%；1.72 kPa: 0.877±0.004 , RSD=0.5%; 3.36 kPa: 1.219±0.018 , RSD=1.5%; 5.12 kPa: 1.544±0.020 , RSD=1.3%; 6.76 kPa: 1.887±0.023 , RSD=1.2%; 8.11 kPa: 2.340±0.017 , RSD=0.7%; 10.17 kPa: 2.485±0.046 , RSD=1.9%; 12.79 kPa: 2.778±0.029 , RSD=1.0%; 16.17 kPa: 2.893±0.031 , RSD=1.1%; 20.43 kPa: 3.300±0.030 , RSD=0.9%; 26.7 kPa: 2.658±0.004 , RSD=0.2%
0.3 wt.%	1.05 kPa: 0.492±0.007 , RSD=1.4%；2.65 kPa: 0.967±0.024 , RSD=2.5%; 4.53 kPa: 1.510±0.020 , RSD=1.3%; 7.13 kPa: 2.033±0.012 , RSD=0.6%; 9.16 kPa: 2.493±0.020 , RSD=0.8%; 12.01 kPa: 2.895±0.025 , RSD=0.9%; 14.70 kPa: 4.098±0.049 , RSD=1.2%; 18.62 kPa: 4.452±0.028 , RSD=0.6%; 22.10 kPa: 4.318±0.060 , RSD=1.4%; 27.12 kPa: 4.685±0.049 , RSD=1.0%
0.5 wt.%	0.81 kPa: 0.516±0.016 , RSD=3.1%；1.84 kPa: 1.236±0.025 , RSD=2.0%; 3.16 kPa: 2.167±0.037 , RSD=1.7%; 4.95 kPa: 2.924±0.067 , RSD=2.3%; 7.47 kPa: 3.677±0.039 , RSD=1.1%; 9.68 kPa: 4.737±0.088 , RSD=1.9%; 12.37 kPa: 5.514±0.107 , RSD=1.9%; 15.53 kPa: 6.194±0.110 , RSD=1.8%; 18.62 kPa: 7.205±0.149 , RSD=2.1%; 21.81 kPa: 8.111±0.082 , RSD=1.0%; 26.17 kPa: 8.789±0.158 , RSD=1.8%
0.7 wt.%	0.83 kPa: 0.574±0.012 , RSD=2.1%；1.72 kPa: 1.280±0.013 , RSD=1.0%; 3.04 kPa: 2.081±0.015 , RSD=0.7%; 4.78 kPa: 2.615±0.039 , RSD=1.5%; 7.23 kPa: 3.027±0.029 , RSD=1.0%; 9.95 kPa: 3.129±0.038 , RSD=1.2%; 12.30 kPa: 3.472±0.078 , RSD=2.2%; 15.07 kPa: 4.055±0.061 , RSD=1.5%; 18.03 kPa: 4.802±0.058 , RSD=1.2%; 21.56 kPa: 5.173±0.097 , RSD=1.9%; 25.43 kPa: 4.755±0.067 , RSD=1.4%
0.9 wt.%	0.74 kPa: 0.573±0.024 , RSD=4.2%；1.72 kPa: 0.901±0.013, RSD=1.4%; 3.33 kPa: 1.998±0.014 , RSD=0.7%; 5.64 kPa: 2.570±0.043 , RSD=1.7%; 8.89 kPa: 2.885±0.030 , RSD=1.0%; 12.08 kPa: 3.073±0.027 , RSD=0.9%; 17.00 kPa: 3.330±0.055 , RSD=1.7%; 20.95 kPa: 3.233±0.036 , RSD=1.1%; 26.09 kPa: 3.292±0.065 , RSD=2.0%

The minimum compressive stress detected is: 0.25 kPa, with a corresponding relative change in induced voltage of zero. The relative standard deviation (RSD) for all the data points mentioned above is less than 10.0%, demonstrating good repeatability and stability.

Continue Note S5: Table S4: Reproducibility analysis of the extracted optimal sensitivity data points (n=5).

0.5 wt.%	0.81 kPa: 0.516±0.016 , RSD=3.1%； 1.84 kPa: 1.236±0.025 , RSD=2.0%; 3.16 kPa: 2.167±0.037 , RSD=1.7%; 4.95 kPa: 2.924±0.067 , RSD=2.3%; 7.47 kPa: 3.677±0.039 , RSD=1.1%; 9.68 kPa: 4.737±0.088 , RSD=1.9%; 12.37 kPa: 5.514±0.107 , RSD=1.9%; 15.53 kPa: 6.194±0.110 , RSD=1.8%; 18.62 kPa: 7.205±0.149 , RSD=2.1%; 21.81 kPa: 8.111±0.082 , RSD=1.0%; 26.17 kPa: 8.789±0.158 , RSD=1.8%

The relative standard deviation (RSD) for all the data points mentioned above is less than 10.0%, demonstrating good repeatability and stability.

Continue Note S5: Table S5: Reproducibility analysis of the conductivity graph (n=3).

0 wt.%	0.0540±0.0023 S/m, RSD=4.3%
0.1 wt.%	0.0181±0.0004 S/m, RSD=2.2%
0.3 wt.%	0.0297±0.0003 S/m, RSD=1.0%
0.5 wt.%	0.0125±0.0003 S/m, RSD=2.4%
0.7 wt.%	0.0352±0.0028 S/m, RSD=8.0%
0.9 wt.%	0.0663±0.0039 S/m, RSD=5.9%

The relative standard deviation (RSD) for all the data points mentioned above is less than 10.0%, demonstrating good repeatability and stability.

Continue Note S5: Table S6: Analysis of reproducibility for plots of relative change rate of induced charge (n=3).

0 wt.%	0.86 kPa: 0.0126±0.0002 nC, RSD=1.6%; 3.11 kPa: 0.0360±0.0018 nC, RSD=5.0%; 6.00 kPa: 0.0546±0.0012 nC, RSD=2.2%; 10.54 kPa: 0.0834±0.0036 nC, RSD=4.3%; 14.26 kPa: 0.1055±0.0041 nC, RSD=3.9%; 19.11 kPa: 0.1248±0.0053 nC, RSD=4.2%;25.48 kPa: 0.1433±0.0061 nC, RSD=4.3%
0.1 wt.%	0.74 kPa: 0.0197±0.0012 nC, RSD=6.1%; 1.96 kPa: 0.0434±0.0020 nC, RSD=4.6%; 4.17 kPa: 0.0634±0.0019 nC, RSD=3.0%; 6.86 kPa: 0.0869±0.0030 nC, RSD=3.5%; 11.27 kPa: 0.1106±0.0041 nC, RSD=3.7%; 16.66 kPa: 0.1352±0.0049 nC, RSD=3.6%;21.56 kPa: 0.1658±0.0063 nC, RSD=3.8%; 25.73 kPa: 0.1788±0.0071 nC, RSD=4.0%
0.3 wt.%	1.27 kPa: 0.0104±0.0002 nC, RSD=1.9%; 3.43 kPa: 0.0258±0.0004 nC, RSD=1.6%; 6.49 kPa: 0.0511±0.0007 nC, RSD=1.4%; 11.17 kPa: 0.0657±0.0006 nC, RSD=0.9%; 15.19 kPa: 0.0799±0.0007 nC, RSD=0.9%; 20.26 kPa: 0.0983±0.0020 nC, RSD=2.0%;25.24 kPa: 0.1061±0.0015 nC, RSD=1.4%
0.5 wt.%	1.57 kPa: 0.0075±0.0002 nC, RSD=2.7%; 3.92 kPa: 0.0145±0.0003 nC, RSD=2.1%; 7.35 kPa: 0.0233±0.0014 nC, RSD=6.0%; 11.52 kPa: 0.0341±0.0007 nC, RSD=2.1%; 16.66 kPa: 0.0402±0.0008 nC, RSD=2.0%; 20.58 kPa: 0.0431±0.0010 nC, RSD=2.3%;25.77 kPa: 0.0471±0.0004 nC, RSD=0.8%
0.7 wt.%	1.25 kPa: 0.0084±0.0008 nC, RSD=9.5%; 4.48 kPa: 0.0161±0.0013 nC, RSD=8.1%; 7.42 kPa: 0.0209±0.0016 nC, RSD=7.7%; 12.05 kPa: 0.0508±0.0027 nC, RSD=5.3%; 15.68 kPa: 0.0820±0.0041 nC, RSD=5.0%; 20.58 kPa: 0.1315±0.0062 nC, RSD=4.7%;25.87 kPa: 0.1514±0.0072 nC, RSD=4.8%
0.9 wt.%	1.59 kPa: 0.0009±0.000008 nC, RSD=0.9%; 3.58 kPa: 0.0139±0.0002 , RSD=1.4%; 6.71 kPa: 0.0352±0.0010 , RSD=2.8%; 11.64 kPa: 0.0577±0.0007 , RSD=1.2%; 13.74 kPa: 0.0596±0.0007 , RSD=1.2%; 17.57 kPa: 0.0629±0.0010 , RSD=1.6%;22.05 kPa: 0.0353±0.0007 , RSD=2.0%; 25.97 kPa: 0.0286±0.0005 , RSD=1.7%

The minimum compressive stress detected is: 0.25 kPa, with a corresponding relative change in induced voltage of zero. The relative standard deviation (RSD) for all the data points mentioned above is less than 10.0%, demonstrating good repeatability and stability.

Continue Note S5: Table S7: Analysis of repeatability in the graphical representation of the relative change rate of total capacitance (n=3).

0 wt.%	1.23 kPa: 0.271±0.024 , RSD=8.9%； 2.45 kPa: 0.602±0.052 , RSD=8.6%; 4.9 kPa: 0.684±0.026 , RSD=3.8%; 7.35 kPa: 1.133±0.021 , RSD=1.9%; 12.25 kPa: 1.676±0.064 , RSD=3.8%; 19.6 kPa: 1.754±0.078 , RSD=4.4%;25.24 kPa: 2.537±0.084 , RSD=3.3%
0.1 wt.%	1.23 kPa: 1.118±0.105 , RSD=9.4%； 2.45 kPa: 2.762±0.146 , RSD=5.3%; 4.9 kPa: 3.351±0.330 , RSD=9.8%; 7.35 kPa: 4.282±0.391 , RSD=9.1%; 12.25 kPa: 5.197±0.004 , RSD=0.1%; 19.6 kPa: 5.595±0.351 , RSD=6.3%;25.24 kPa: 7.105±0.100 , RSD=1.4%
0.3 wt.%	1.23 kPa: 1.910±0.059 , RSD=3.1%； 2.45 kPa: 3.002±0.093 , RSD=3.1%; 4.9 kPa: 4.820±0.156 , RSD=3.2%; 7.35 kPa: 5.458±0.169 , RSD=3.1%; 12.25 kPa: 6.009±0.440 , RSD=7.3%; 19.6 kPa: 6.730±0.191 , RSD=2.8%;25.24 kPa: 7.096±0.354 , RSD=5.0%
0.5 wt.%	1.23 kPa: 9.548±0.507 , RSD=5.3%； 2.45 kPa: 12.619±0.265 , RSD=2.1%; 4.9 kPa: 13.992±0.655 , RSD=4.7%; 7.35 kPa: 14.556±0.509 , RSD=3.5%; 12.25 kPa: 14.794±0.864 , RSD=5.8%; 19.6 kPa: 14.214±0.499 , RSD=3.5%;25.24 kPa:14.571±0.989 , RSD=6.8%
0.7 wt.%	1.23 kPa: 1.340±0.081 , RSD=6.0%； 2.45 kPa: 1.804±0.079 , RSD=4.4%; 4.9 kPa: 2.937±0.112 , RSD=3.8%; 7.35 kPa: 3.351±0.080 , RSD=2.4%; 12.25 kPa: 3.714±0.307 , RSD=8.3%; 19.6 kPa: 4.538±0.265 , RSD=5.8%;25.24 kPa: 5.464±0.008 , RSD=0.1%
0.9 wt.%	1.23 kPa: 1.277±0.023 , RSD=1.8%； 2.45 kPa: 1.756±0.032 , RSD=1.8%; 4.9 kPa: 3.194±0.211 , RSD=6.6%; 7.35 kPa: 3.617±0.083 , RSD=2.3%; 12.25 kPa: 3.244±0.032 , RSD=1.0%; 19.6 kPa: 3.510±0.109 , RSD=3.1%;25.24 kPa: 4.308±0.099 , RSD=2.3%

The minimum compressive stress detected is: 0.25 kPa, with a corresponding relative change in induced voltage of zero. The relative standard deviation (RSD) for all the data points mentioned above is less than 10.0%, demonstrating good repeatability and stability.

Continue Note S5: Table S8: Repeatability analysis of signal pulse width extracted from preliminary contact recognition experiments (n=3).

Smooth copper	65.0±2.6 ms, RSD=4.0%
Rough copper	244.6±7.7 ms, RSD=3.1%
Skin	299.0±11.7 ms, RSD=3.9%
PMMA	364.0±8.0 ms, RSD=2.2%
Kapton	344.3±5.5 ms, RSD=1.6%
PTFE	645.3±17.2 ms, RSD=2.7%

The relative standard deviation (RSD) for all the data points mentioned above is less than 10.0%, demonstrating good repeatability and stability.

Continue Note S5: Table S9: 10,000-cycle test data (0.5 wt.%, 3 Hz, 3.5 kPa) supplementary and reproducibility analysis.

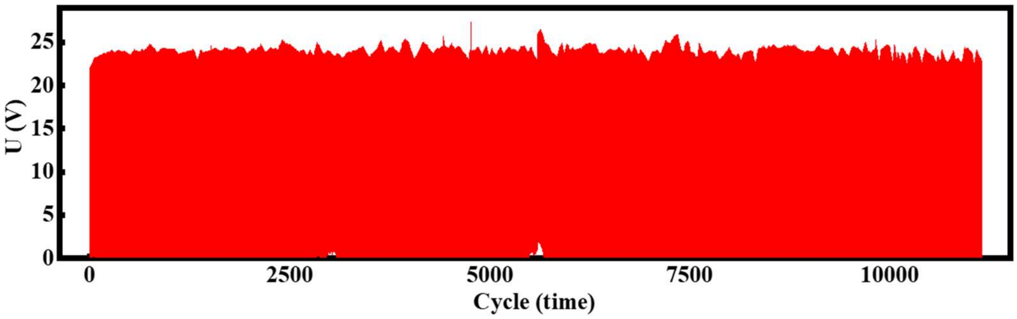

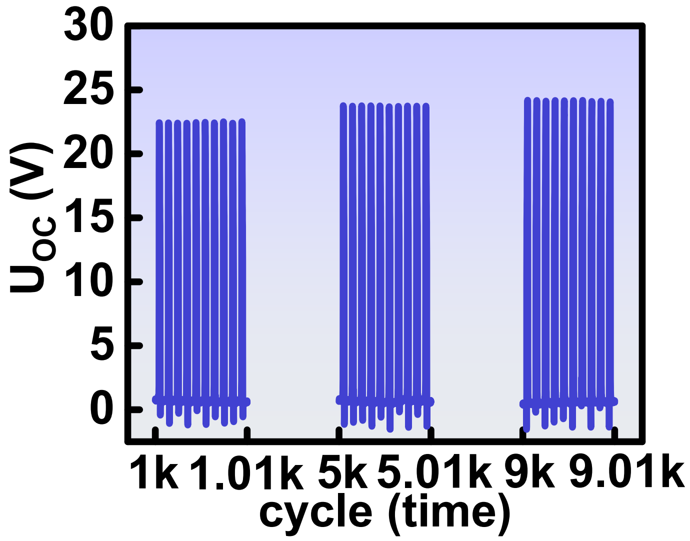

1k-1.01k<br>(n=10)	Sample upper limit: $\overline{x}$=22.441 V, σ=0.043, RSD=0.2%;<br>Sample lower limit: $\overline{x}$=0.670 V, σ=0.028, RSD=4.2%;
5k-5.01k<br>(n=10)	Sample upper limit: $\overline{x}$=23.734 V, σ=0.025, RSD=0.1%;<br>Sample lower limit: $\overline{x}$=0.689 V, σ=0.041, RSD=6.0%;
9k-9.01k<br>(n=10)	Sample upper limit: $\overline{x}$=24.141 V, σ=0.039, RSD=0.2%;<br>Sample lower limit: $\overline{x}$=0.650 V, σ=0.051, RSD=7.8%;
Overall statistics	Overall upper limit: $\overline{x}$=23.439 V, σ=0.887, RSD=3.8%;<br>Overall lower limit: $\overline{x}$=0.669 V, σ=0.019, RSD=2.8%;

The relative standard deviation (RSD) for all the data points mentioned above is less than 10.0%, demonstrating good repeatability and stability.

From the statistical results above: the overall signal upper limit of the cyclic test is 23.439 V (denoted as $\overline{x}_h$), and the overall signal lower limit is 0.669 V (denoted as $\overline{x}_l$).

The response signal-to-noise ratio (SNR) can be calculated as SNR=$20\log_{10}\frac{\bar{x}_h}{\bar{x}_l}$=30.9 dB ＞20 dB, which meets the signal detection requirement.

Supporting pictures

Note S6: The source and morphology of the graphene used in the experiment.

Purity: >95%

Thickness: 3.4-8 nm

Layer size: 5-50 μm

Number of floors: 6 to 10 floors

Conductivity: 10^5 S/m

Oxygen content: 0.5%

Sulfur content: 0.5%

Metal impurity content: 100 ppm

Specific surface area: 100-300 m ² /g

SEM：

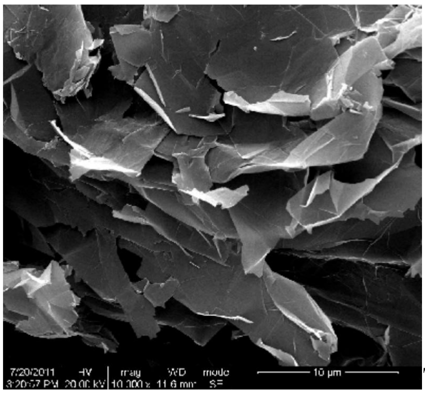

TEM：

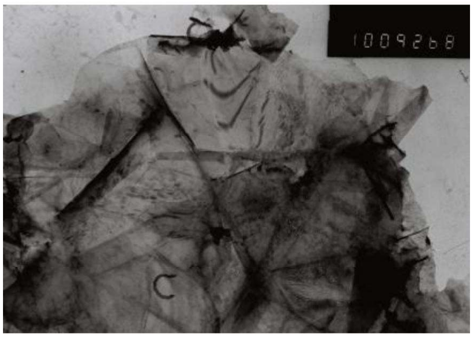

AFM：

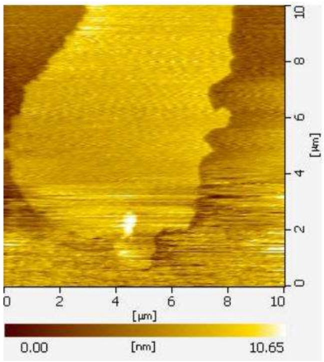

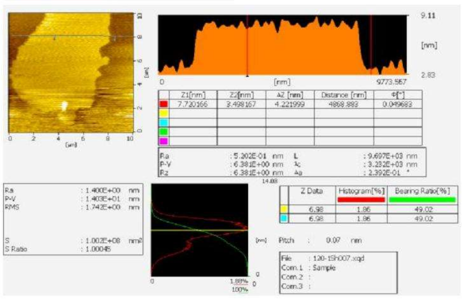

XRD：

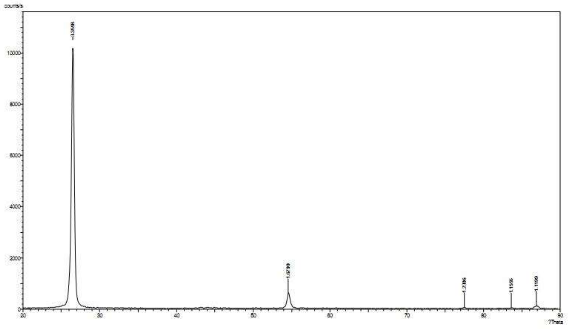

Note S7: The dispersion principle and method of the G and the potential impact of the surfactant.

The dispersion principle of the G. Sodium dodecylbenzenesulfonate (SDBS) is used as a dispersant to disperse graphene mainly through the electrostatic stabilization mechanism. Its working principle is as follows: SDBS is an ionic dispersant that is anchored on the graphene surface through physical adsorption or chemical bonding. After dissociation, it causes the particle surface to become charged, forming a double electric layer. According to the DLVO theory, the repulsive energy of the double electric layer is proportional to the square of the surface potential. When pH = 9, SDBS can make the Zeta potential of the graphene surface reach -45 mV, forming a stable electrostatic repulsion layer. At this time, the aggregate size can be reduced to below 200 nm.

The dispersion method of the G. Dissolve the corresponding amount of dispersant in the liquid medium. This process can usually be achieved by shaking, low-speed stirring or ultrasonic treatment. The duration of shaking, ultrasonic treatment or stirring is determined by the experimental temperature and the solubility of the corresponding dispersant. Generally, it won't take a long time, about 15 minutes (at room temperature of 25 degrees Celsius). Then, slowly add the nanomaterial (powder) to the solvent where the dispersant has been dissolved. You can add it in several portions until all is added. Next, add the aforementioned mixture to a grinding tank or sanding tank for grinding or sanding. Finally, test the fineness and filtration.

The potential impact of the surfactant.

SDBS, an anionic dispersant, contains sulfonate groups in its molecules that are highly hydrophilic and carry negative charges. If residual SDBS remains in the hydrogel network, it can enhance hydrophilicity and improve ionic conductivity. However, the residual SDBS may act as an "impurity" that interferes with the cross-linking of the hydrogel polymer network, potentially exerting subtle effects on the long-term stability or mechanical strength of the matrix. In addition, SDBS anchors onto the graphene surface through physical adsorption or chemical bonding, forming a negatively charged coating layer (with a zeta potential of approximately -45 mV). This coating acts as an

"intermediate layer" between graphene and the polymer matrix, effectively preventing graphene reaggregation, promoting its uniform dispersion in the polymer, and reducing interfacial defects, thereby benefiting stress transfer and electron/ion transport. However, if the coating layer is too thick or not adequately removed, it may introduce additional interfacial resistance between graphene and the matrix, potentially hindering direct electron tunneling, reducing the intrinsic electronic conductivity of the composite material, and introducing additional interfacial polarization, which could affect the dielectric constant and loss.

Note S8: The macroscopic and cross-sectional SEM images of the freeze-dried GH.

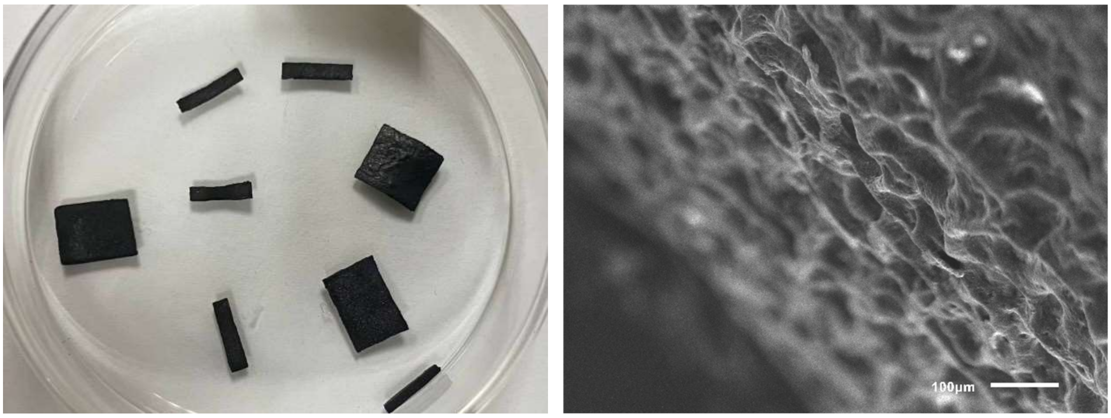

Note S9: The macrograph of the 0.5 wt.% GH monolith.

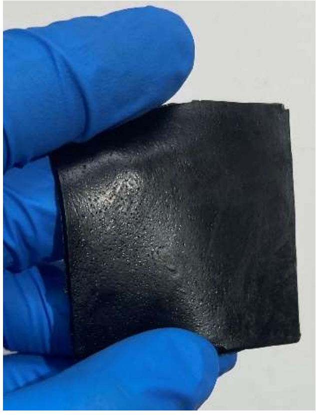

Note S10: The photograph of the SGHS sample from the 0.5 wt.% GH.

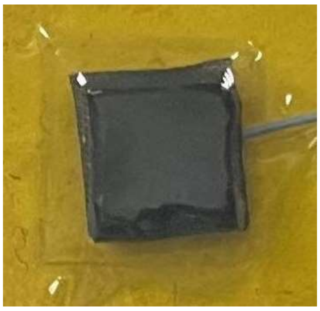

Note S11: The COMSOL simulation of electric potential distribution during a single contact-separation cycle.

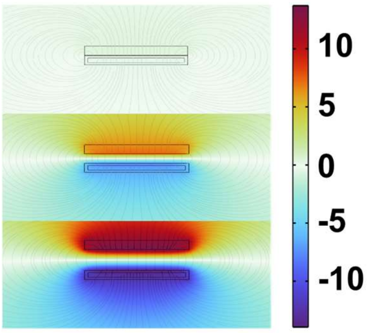

Supplement: Supplementary file 1 [file nanomaterials-16-00453-s001.zip › nanomaterials-4234222-supplementary.pdf]
